# Segmented Linear Regression Models for Assessing Change in Retrospective Studies in Healthcare

**DOI:** 10.1155/2019/9810675

**Published:** 2019-01-22

**Authors:** Epaminondas Markos Valsamis, David Ricketts, Henry Husband, Benedict Aristotle Rogers

**Affiliations:** ^1^Brighton and Sussex University Hospitals NHS Trust, Trauma and Orthopaedic Department, Brighton BN2 5BE, UK; ^2^Faculty of Mathematics, University of Cambridge, Cambridge CB3 0WA, UK

## Abstract

**Introduction:**

In retrospective studies, the effect of a given intervention is usually evaluated by using statistical tests to compare data from before and after the intervention. A problem with this approach is that the presence of underlying trends can lead to incorrect conclusions. This study aimed to develop a rigorous mathematical method to analyse temporal variation and overcome these limitations.

**Methods:**

We evaluated hip fracture outcomes (time to surgery, length of stay, and mortality) from a total of 2777 patients between April 2011 and September 2016, before and after the introduction of a dedicated hip fracture unit (HFU). We developed a novel modelling method that fits progressively more complex linear sections to the time series using least squares regression. The method was used to model the periods before implementation, after implementation, and of the whole study period, comparing goodness of fit using *F*-tests.

**Results:**

The proposed method offered reliable descriptions of the temporal evolution of the time series and augmented conclusions that were reached by mere group comparisons. Reductions in time to surgery, length of stay, and mortality rates that group comparisons would have credited to the hip fracture unit appeared to be due to unrelated underlying trends.

**Conclusion:**

Temporal analysis using segmented linear regression models can reveal secular trends and is a valuable tool to evaluate interventions in retrospective studies.

## 1. Introduction

The National Health Service has a strong culture of quality improvement [1]. A good evidence base is needed to drive this process, and these data are often derived from retrospective studies and audits [2].

**Figure 1:**
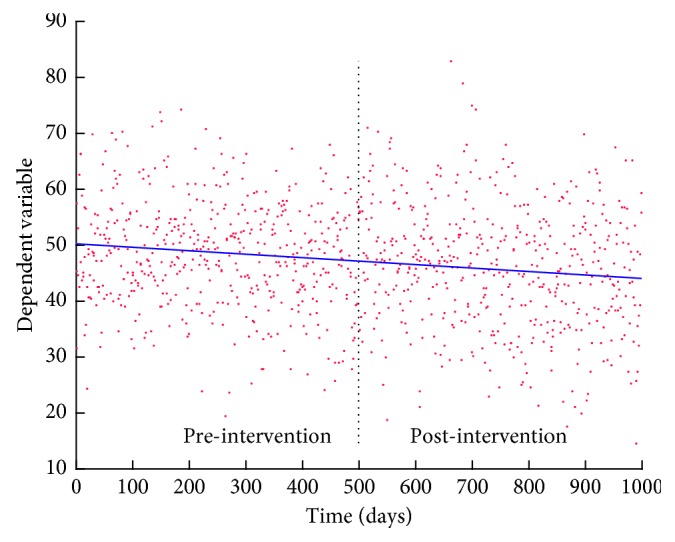
Gaussian noise superimposed on a mildly decreasing trend highlights a situation where the mean of pre-intervention measure is significantly different from that of the post-intervention measure (*p*=0.0002) when the change is entirely due to the trend and not the intervention. The dashed vertical line separates the two groups. The blue line represents the line of regression of the mean value of the simulated variable.

An important part of the audit cycle is comparison of data before and after a given intervention [3]. Typically, mean values or ranks of outcome measures before and after the intervention are compared, and differences are tested statistically for significance. This method assumes stationarity of data which is often not the case. Nonstationarity of data is difficult to distinguish from causal change, and group comparisons are unable to distinguish underlying (secular) trends from intervention-induced change. This can lead to erroneous conclusions [4–6] and is illustrated by a simulated example shown in Figure 1. This shows a hypothetical intervention that made no contribution to a change. Group comparison would attribute this to the intervention (*p*=0.0002). While this is obvious in this example, more subtle trends obscured by highly variable data are more challenging to identify. Researchers attempt to limit the influence of trends by selecting data closer to the point of the intervention, but this does not annul the effect of a secular trend when one is present [6]. It also sacrifices much of the data, although it should be noted that data spanning long time intervals are more susceptible to confounding parameters that are external to the purpose of the study.

Alternative approaches that accommodate trends use interrupted linear or higher-order regression, but these suffer from bias because the interruption is chosen at the point of the intervention [7, 8]. Randomised controlled trials avoid many of these issues but are often expensive and time consuming. Consequently, any method that can improve the reliability of retrospective studies has wide potential application to the wealth of data that such studies make available at relatively low cost.

The aim of this study was to describe a novel method of modelling the temporal variation of a time series of data to help identify secular trends in retrospective studies. Such a method should reliably reveal underlying trends spanning the entire study period, without bias to the intervention. Importantly, there should be a mathematically robust technique to determine whether a trend in the data is significant or due to random variation. We developed and applied a novel method to analyse the temporal variation in hip fracture outcomes in a retrospective study that aimed to evaluate the implementation of a dedicated hip fracture unit (HFU).

## 2. Methods

### 2.1. Hip Fracture Unit

In July 2015, a level 1 Major Trauma Centre (MTC) in the United Kingdom established a dedicated HFU to free up beds within the MTC. The HFU was located in a nearby district general hospital. Services were reconfigured to include ambulance triage, daily consultant-led dedicated theatre lists for proximal femoral fractures, and coordination of the necessary multidisciplinary team of staff at the district hospital. These changes were all instituted simultaneously on July 1^st^, 2015.

### 2.2. Study Group

We studied 2777 patients who sustained proximal femoral fragility fractures. Of these, 2117 patients (2176 fractures) sustained their fractures prior to commencement of the HFU (study period April 2011–June 2015), and 660 patients (671 fractures) were treated after the introduction of the HFU (study period July 2015–September 2016).

### 2.3. Data

From a retrospective review of patient notes, the following data were obtained:

1. Time to surgical intervention (hours from time of admission)
2. Median length of hospital stay in days (LOS)
3. Mortality rate at 30 days, 120 days, and 365 days

Patient demographic data were also collected. Both hospitals were part of the same trust, and as such the same sources of data collection were used: “e-Oasis” and “Symphony” databases.

### 2.4. Statistical Analysis

Data before and after the intervention (introduction of the HFU) were compared. The data were analysed using both conventional statistical tests and temporal analysis using a novel model-fitting method. MATLAB (Natick, MA, USA) was used to apply segmented least squares regression to determine parameters of the nested models that are fitted to the time series [9, 10]. The models were compared for goodness of fit using *F*-tests to determine the best-fitting model. The large number of data points (*n* > 2800) helped overcome limitations arising from the non-normality of data to validate the use of *F*-tests to compare nested models [11].

For conventional statistical tests, continuous variables (time to surgery and length of stay) were compared using Mann–Whitney tests while categorical data (30-day, 120-day, and 365-day mortality rates) were compared using Fisher's exact tests. Demographic data were compared using Mann–Whitney tests (age) and chi-squared tests (gender, pathological fracture, ASA grade, and re-operation). These tests were applied using the MedCalc software suite (Ostend, Belgium) [12]. Statistical significance was set at *p* < 0.05.

### 2.5. Segmented Linear Regression Model Fitting

In this study, we used an adaptation of a segmented linear regression technique previously published by one of the authors (EMV) in the context of learning curve modelling [9, 13].

We used least squares regression to fit a set of four progressively more complex models to data of the time series. Our models ranged from a simple plateau to two adjoining straight lines (linear splines) with a single knot at the point in time that minimised the sum of the square of residuals. This range of models accommodates a respectable degree of complexity that can capture changing trends in the study period but also maintains simplicity and meaningfulness in the descriptions of trends.

The four models, in increasing order of complexity, are as follows:

(i) *Plateau model.* This is the simplest model. It is a horizontal line (plateau) at the average value of the outcome measure over the study period.
  (1) $$\begin{array}{c} y=\bar{y}, \end{array}$$
  where $\bar{y}=\sum_{i}y_{i}/n$.
(ii) *Line model.* The next model is a straight line determined by least squares regression of the variable (*y*) on the time (*t*):
  (2) $$\begin{array}{c} y=mt+c, \end{array}$$
  where *m*=∑_*i*_*t*_*i*_*y*_*i*_/∑_*i*_*t*_*i*_^2^ is the gradient and *c*=(∑_*i*_*y*_*i*_/*n*) − (*m*∑_*i*_*t*_*i*_/*n*) is the *y*-axis intercept.
(iii) *Line-plateau (or plateau-line) model.* This model is made up of two sections: a straight line adjoining a horizontal plateau at the *k*th value of *t*(*t*=*t*_*k*_) or vice versa. The parameters of each section (gradient and intercept) are determined by least squares regression while the adjoining point (*t*=*t*_*k*_) is determined as that which minimises the sum of squares of residuals (Appendix A).
The line-plateau model is given by *y*_*i*_ *=* *m*_*k*_*t*_*i*_ *+* *c*_*k*_ for *i* *=* 1 to *k* and *y*_*j*_ *=* *Y*_k_ for *j* *=* *k* + 1 to *n* where *m*_*k*_ and *c*_*k*_ are the gradient and *y*-axis intercept of the straight line, respectively, and *Y*_*k*_ the value of the plateau.
The plateau-line model is given by *y*_*i*_ *=* *c*_k_*for i* *=* *1 to k and y*_*j*_ *=* *m*_*k*_ (*t*_*j*_ – *t*_*k*_) *+* *c*_*k*_ for *j* *=* *k* + 1 to *n* where *m*_*k*_ is the gradient of the straight line and *c*_*k*_ the value of the plateau.
(iv) *Line-line model.* This model is made up of two sections: a straight line adjoining a second straight line at the *k*th value of *t* (*t* *=* *t*_*k*_). The two straight lines are determined by least squares regression of the variable (*y*) on the time (*t*) using a closed form. The adjoining point (*t* *=* *t*_*k*_) is determined as that which minimises the sum of squares of residuals (Appendix B).

The resulting lines have equations *y*_*i*_ *=* *m*1_*k*_*t*_*i*_ *+* *c*_*k*_ (*i* *=* 1 to *k*) and *y*_*j*_ *=* *m*2_*k*_ (*t*_*j* _– *t*_*k*_) *+* *y*_*k*_ (*j* *=* *k* + 1 *to n*) where *m*1_*k*_ and *c*_*k*_ are the gradient and *y*-axis intercept of the first line and *m*2_*k*_ is the gradient of the last straight line.

After all four models have been fitted, we select the best model as the simplest one unless a more complex one fits the data significantly better. Significance is confirmed by *F*-tests that compare the sum of the squares of residuals between models. A simple tabular method listing the *p* values of *F*-tests conducted pairwise between all four models helps select the best model. This method is illustrated in Table 1. This technique (*F*-test as applied to nested models) accounts for and prevents, the risk of overfitting whereby higher-order models (e.g., line-line) will always demonstrate improved fit, but at the expense of additional parameters [14].

### 2.6. Application of the Method to the Study

Our proposed method for selecting the best model was applied over three distinct periods of time: (i) before the intervention (pre-HFU), (ii) after the intervention (post-HFU), and (iii) the entire time series (including both pre-HFU and post-HFU periods). The model describing the entire time series was subsequently compared to the models for the separate pre-HFU and post-HFU periods using *F*-tests to determine whether separate models offered a significantly better fit.

## 3. Results

### 3.1. Time to Surgery

Conventional tests: median time to surgical intervention decreased after introduction of the HFU. This change was not significant (21.51 hours pre-HFU to 20.75 hours post-HFU, Mann–Whitney test: *p*=0.150).

**Figure 2:**
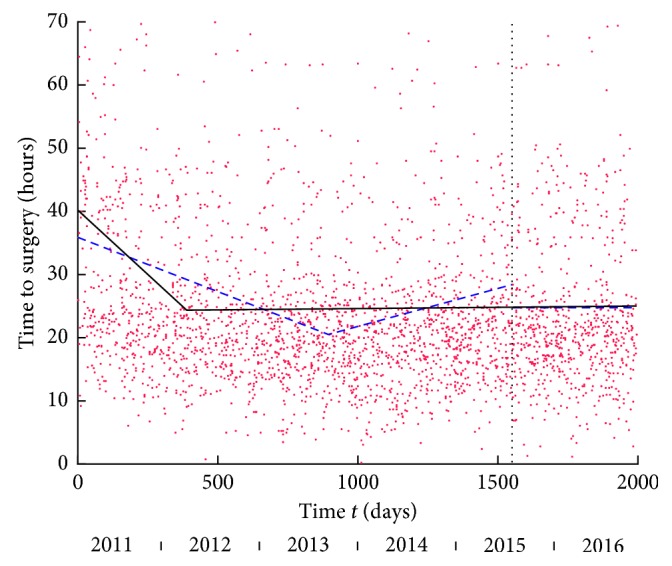
Time to surgery versus time *t* in days from the start of the study. The vertical dashed line marks the onset of the HFU. The black line shows line-plateau as best-fitting linear model for entire period: the line has equation *y* = −0.0414*t* + 40.1868; plateau at *y* = 24.7033 reached after 375 days. Dashed blue lines are best-fitting linear models for pre-HFU and post-HFU periods. For pre-HFU period, the best model in line-line model with first line *y* = −0.0172*t* + 35.8863 and second line *y* = 0.0120 (*t* − 894) + 20.5137 with delimiting time 894 days. For post-HFU period, the best model is the plateau at *y* = 24.7359.

Temporal analysis: the best-fitting model for the entire data set was the line-plateau (Figure 2), illustrating that time to surgery decreased during the initial period of the study, well before the introduction of the HFU. The line‐line model was the best model for the period pre-HFU and showed a decreasing trend followed by an increasing trend. The best model post-HFU was the plateau, taking a value at about the average plateau of the line-plateau model fitted on the entire study period. Separate models for the periods before and after the intervention did not offer a better fit compared to using a single model for the entire time series (*p*=0.100). As a result, a single model over the entire period offered the best temporal description, suggesting that a decrease in the time to surgery occurred early in the study period, well before the intervention.

### 3.2. Length of Stay

Conventional tests: median length of stay (LOS) decreased after introduction of the HFU, though not significantly (15 days pre-HFU to 14 days post-HFU, Mann–Whitney test: *p*=0.410).

**Figure 3:**
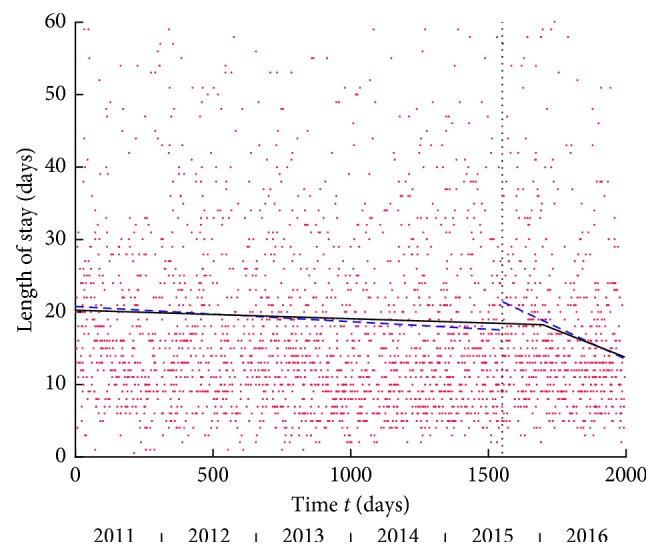
Length of stay versus time *t* in days from the start of the study. The vertical dashed line marks the onset of the HFU. The black line shows the line-line model as the best-fitting linear model for entire time period: first line *y* = −0.0012*t* + 20.2556 and second line *y* = −0.0150 (*t* − 1698) + 18.2532 with delimiting time at 1698. The dashed blue lines are the best-fitting linear models for the pre-HFU and post-HFU periods. The best model for the pre-HFU period is the single line with equation *y* = –0.0021*t* + 20.7696, and best model for post-HFU is the single line with equation *y* = –0.01769 (*t* − 1550) + 48.8532.

Temporal analysis: the best-fitting model for the entire time series was the line-line model (Figure 3). It demonstrated a slowly decreasing rate in LOS followed by a sharply decreasing rate in LOS that started about half a year after the introduction of the HFU. The onset of this sharp change in the rate explained the difference in median LOS pre- and post-HFU. Separate pre-HFU analysis and post-HFU analysis demonstrated the single line to be the best model in both. Separate models offered a better fit than the overall model (*p*=0.017) confirming that the intervention (HFU) contributed to change by causing an acceleration in the decrease of LOS.

### 3.3. 30-Day Mortality

Conventional tests: 30-day mortality rate decreased significantly after introduction of the HFU (5.47% pre-HFU to 3.13% post-HFU, Fisher's Exact test: *p*=0.014).

**Figure 4:**
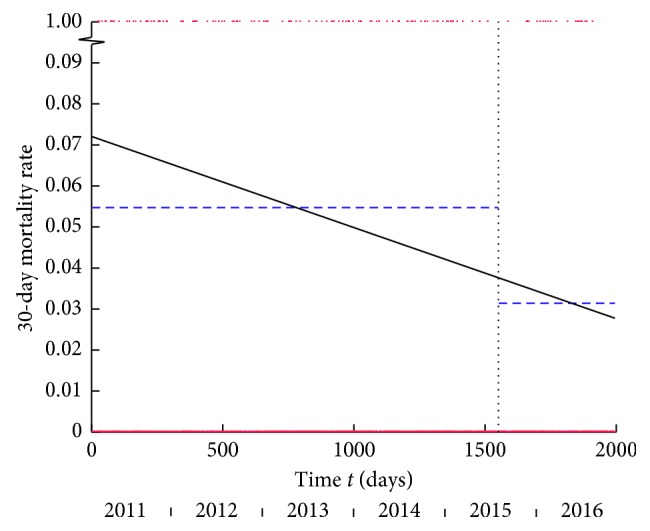
30-day mortality versus time *t* in days from the start of the study. The vertical dashed line marks the onset of the HFU. The single line (black) is the best-fitting linear model for the entire period: equation *y* = −0.00002*t* + 0.0720. The dashed blue lines show plateaus are the best-fitting linear models for the pre-HFU (*y* = 0.0547) and post-HFU (*y* = 0.0314) periods. Data points 0 or 1 for dead or alive in red.

Temporal Analysis: The single line model was the best model when analysing the entire time period, suggesting that 30-day mortality rate followed a declining trend throughout the study period (Figure 4). Separate pre-HFU and post-HFU analysis demonstrated that the plateau fit best in both. Separate models did not offer a better fit compared to the overall model (*p*=1.000). We concluded that 30-day mortality most probably followed a declining trend over the entire study period which appeared to be responsible for the significant reduction found using group comparison. This means that the significant reduction in mortality rates from pre-intervention to post-intervention cannot be simply credited to the intervention.

### 3.4. 120-Day Mortality

Conventional tests: 120-day mortality rate decreased, but not significantly, after introduction of the HFU (12.68% pre-HFU to 10.13% post-HFU, Fisher's Exact test: *p*=0.078).

**Figure 5:**
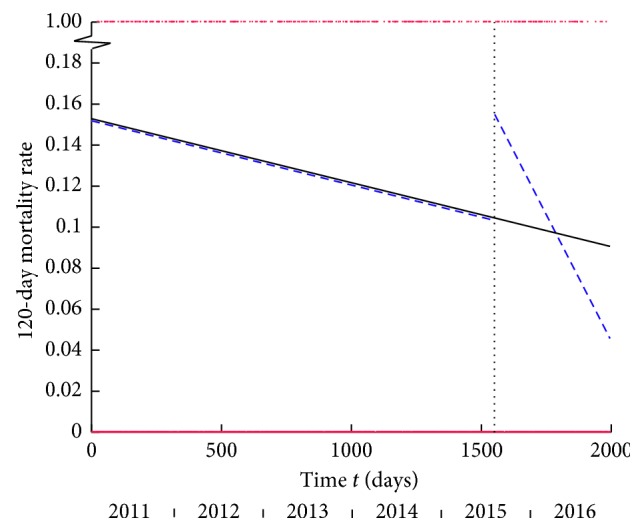
120-day mortality versus time *t* in days from the start of the study. The vertical dashed line marks the onset of the HFU. The black line shows single line as the best-fitting linear model for the entire period: equation *y* = −0.00003*t* + 0.1529. The dashed blue lines are the best fitting linear models for the pre-HFU and post-HFU periods. The best model for the pre-HFU period is the single line with equation *y* = −0.00003*t* + 0.1519, and the best model for the post-HFU period is the single line with equation *y* = −0.00025 (*t*−1550) + 0.1497. Data points 0 or 1 for dead or alive in red.

Temporal analysis: the single line was the best model for the entire time period (Figure 5). Separate pre-HFU and post-HFU analysis demonstrated the line model fit best for both. Separate models did not offer a better fit compared to the model for the entire period (*p*=0.187). The 120-day mortality rate appeared to follow a declining trend that spanned the entire study period, and the HFU did not appear to have caused a reduction in this measure.

### 3.5. 365-Day Mortality

For this outcome measure, post-HFU data are limited to a smaller sample (*n*=316 instead of *n*=677) due to fewer patients having been followed-up.

Conventional Tests: There was a small and non-significant decrease in 365-day mortality (21.46% pre-HFU to 20.57% post-HFU, Fisher's Exact test: *p*=0.769)

**Figure 6:**
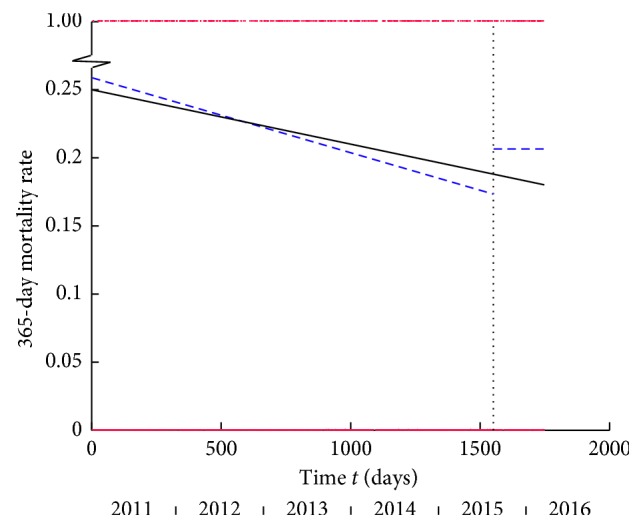
365-day mortality versus time *t* in days from the start of the study. The vertical dashed line marks the onset of the HFU. The black line shows the single line as the best-fitting linear model for the entire period: equation *y* = −0.00004*t* + 0.2497. The dashed blue lines are the best-fitting linear models for the pre-HFU and post-HFU periods. The best model for the pre-HFU period is the single line with equation *y* = −0.00006*t* + 0.2586, and the best model for the post-HFU period is the plateau at *y* = 0.2063. Data points 0 or 1 for dead or alive in red.

Temporal Analysis: The single line model fits the entire time series best (Figure 6). Separate pre-HFU and post-HFU analysis demonstrated the best fit models were the line and plateau, respectively. Separate models did not offer a better fit when compared to the overall model (*p*=0.380). This suggests that a change in one-year mortality was not due to the intervention. Instead, temporal analysis suggested that it followed a decreasing trend that spanned the entire study period. Other causal factors may be implicated in the background changes in 30-day, 120-day, and 365-day mortality rates (Discussion).

### 3.6. Patient Demographics

Patient demographics were analysed before and after implementation of the HFU (Table 2). There were two statistically significant differences: post-HFU, the rate of pathological fracture was less and the distribution of ASA grade was different. These are likely confounding variables which may have also influenced secular trends, further emphasising the importance of temporal analysis in retrospective studies.

## 4. Discussion

In this study, temporal analysis provided important insight into the observed changes before and after the intervention. Importantly, it demonstrated that some of the changes in the outcome measures were likely due to trends that occurred over a longer time period, independent of the intervention.

In modelling the temporal effect of patient mortality, we used simple linear regression instead of logistic regression. We did this because the latter is better suited when dependence on a number of covariates is sought. As we were evaluating the time dependence of mortality rate, simple linear regression offered the advantage of yielding results in a directly interpretable form.

We retrospectively investigated factors that could explain the observed trends as this would help us in evaluating the reliability of the modelling method. We found several factors which are listed below:

1. *Tariff*. The Best Practice Tariff (BPT) was implemented nationwide in April 2010. One criterion was time to surgery within 36 hours (decreased from 48 hours previously) [15]. This could explain the falling trend in time to surgery observed in the early phase of the study in 2011 and 2012.
2. *Orthogeriatrician*. The first orthogeriatrician was appointed to the trust in 2006. Three more have since been appointed and are supported by junior staff.
3. *Implant use*. We discontinued the use of both the dynamic condylar screw (in 2009) and the Austin Moore hemiarthroplasty implant (in 2011). Modular hemiarthroplasty was preferred in our trust after 2010 and was shown to decrease length of stay in a separate study [16].
4. *Clerking proforma*. A dedicated proximal femoral fracture clerking proforma was introduced in 2011 [17].
5. *Theatre prioritisation*. Proximal femoral fractures became prioritised cases on theatre lists in 2011. Prior to this, other cases often took precedence over fractured neck of femur surgery.
6. *Anaesthetic use*. Anaesthetic practice gradually improved, including introduction of a new anaesthetic proforma in 2012 (lower anaesthetic doses and increased use of regional anaesthesia).

The decline in time to surgery that occurred during the initial phase of the study period (long before the HFU) was responsible for the detected drop in median time to surgery from pre-HFU to post-HFU. Temporally, this coincided with the introduction of the Best Practice Tariff in 2010 and introduction of a neck of femur clerking proforma and theatre prioritisation in 2011, which may have contributed to the observed trend. Conventional statistical tests would have misattributed this to the HFU.

Temporal analysis demonstrated that the HFU significantly accelerated the rate of decrease in length of stay, yet this was missed in group comparisons using rank tests. The change in use of hip implants at our trust between 2009 and 2011 has been shown to decrease length of stay [16], possibly explaining the underlying improvement in this variable before the introduction of the HFU. Improvements in anaesthetic use may have also contributed to this.

The underlying decreasing trend in 30-day mortality represented by the single line model over the entire study period and the fact that separate models did not offer a significantly better fit meant that the decrease in 30-day mortality was probably due to a secular trend. Using group comparison tests alone would have misattributed this to the HFU. Temporal analysis of 120-day and 365-day mortality rates similarly demonstrated that an underlying decrease throughout the entire study period was the reason for any observed reduction when using group comparison tests. Interestingly, the post-HFU model for 120-day mortality showed a rapid decline possibly because of a small initial rise subsequently regressing to its normal trend.

The secular trends demonstrating improvement in mortality rate may have been a result of some or all of the aforementioned improvements, although other changes not identified here may have contributed as well.

### 4.1. Evaluation of the Proposed Method

The chosen set of models balance simplicity with the complexity that is required if a single straight line cannot capture underlying trends. The decision to enforce joined segments in models (iii) (line-plateau) and (iv) (line-line) accounted for the expectation that any change within the fitted period is expected to be gradual.

Discontinuity in the variable, such as when an intervention results in a sudden change, can be accommodated by fitting separate models in the phases before and after the intervention. By using linear segments (as opposed to higher-order curves), our models yield meaningful parameters such as plateau values and rates of change. Furthermore, the models are nested which allows statistical comparison with a rigorous tabular method for selection of the best model. The best model offers the most reliable description of the temporal variation of the data.

Our method examined whether the intervention was significant by considering the periods before and after an intervention as separate but also as part of the whole study period. If separate models fit the data significantly better, we concluded that the intervention was significant in bringing change.

### 4.2. Comparison with Other Methods

The simplest form of temporal analysis is a visual inspection of the data of the time series, though this is of little practical value when change is small and obscured by data featuring high variability. Regression analysis is more reliable in revealing the presence of trends and has been used extensively in time series analysis [18].

An adaptation to linear regression is the interrupted time series (ITS) analysis where separate regressions are attempted for the periods before and after the intervention, allowing for discontinuities that could be due to the intervention [19]. This is a well-established method to test the hypothesis that an intervention causes a significant change in the outcome measure over time. However, fitting separate models before and after the intervention biases the method toward finding change at the intervention, and this may not offer the most effective description of how the time series evolved and what secular trends existed (or how these changed) throughout time. Most applications of the ITS method do not compare separate pre- and post-intervention lines with a single model for the entire time period, nor do they envisage using more than one linear segment for each section. This can miss more complex trends [7, 18]. For example, a change in the outcome measure that occurs well before or after the intervention, can be missed by ITS, which would credit the change to the intervention.

**Figure 7:**
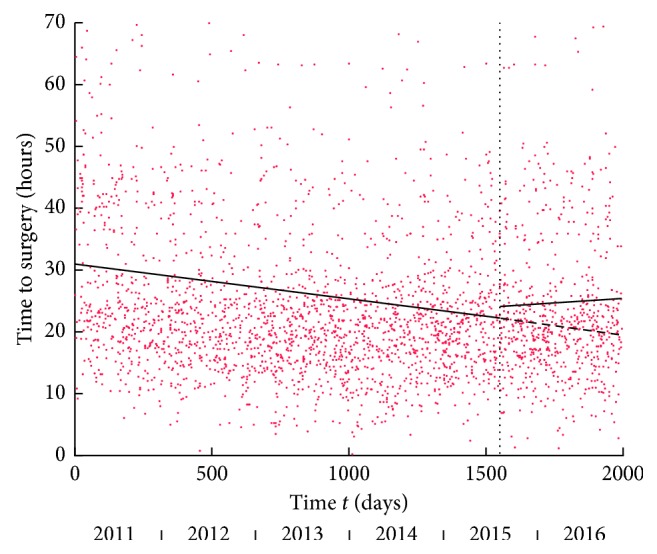
Interrupted time series analysis for time to surgery versus *t* days. The line of best fit pre-HFS (solid black line) was *y* = − 0.0056t + 30.97. Extrapolation of the pre-HFU trend is shown as the dashed line. The line of best fit post-HFU (solid black line) was *y* = 0.0029 (*t* − 1550) + 19.59. Slope change = +0.0085. Intercept change = +1.862. Change in slope: *p*=0.0532; change in intercept: *p*=0.0812.

To contrast our proposed method with the ITS, we applied ITS analysis to one of our outcome variables: the time to surgery (Figure 7). The resulting intercept change and slope change of the ITS at the intervention marginally miss significance (change in slope (*p*=0.0532) and change in intercept (*p*=0.0812)). Nonetheless, as ITS focuses its attention at the intervention, the analysis would have deemed the HFU ineffective while missing the important reduction in time to surgery early in the study. *F*-tests demonstrated significantly improved goodness of fit when using our proposed method, compared to the ITS (*p* < 0.0001). This is not surprising given that our method enabled model change at different time points, yielding a lower sum of the square of residuals. In this scenario (time to surgery), our method concurred with the conclusion when using ITS analysis, but the two methods are fundamentally different, and their conclusions may not agree in all cases. Indeed, our method did not solely evaluate the effect of an intervention (as the ITS does) but described the overall trends within the study period, as well as providing a method to test the significance of change at the intervention.

### 4.3. Limitations

The first limitation of the proposed method is that it cannot capture change that is more complex than envisaged by two adjoining segments, (for example, multiple segmental trends or exponential trends) [18, 20]. In principle, the method can be extended to incorporate three or more segments, but in embracing higher complexity, one risks yielding unnecessary and meaningless information. Expanding to more complex models should be undertaken with caution.

Second, the method requires dedicated computer programming, as most statistical and spreadsheet software do not envisage the fitting of segmented linear regression with variable adjoining points between the segments. Consequently, a basic level of computer programming is required, which limits its use to more advanced healthcare analysts.

Another important limitation is in using *F*-tests to compare the goodness of fit of the various models when residuals do not meet the assumption of being normally distributed [11]. While this may have an effect on the reliability of *p* values that are obtained from the *F*‐distribution, particularly when applying the method to mortality rates, it affects all models equally and should not decrease the effectiveness of the method in selecting the best model. Indeed, setting significance at 5% is imposing an arbitrary threshold which affects model selection, and different significance levels will undoubtedly yield different best models in marginal situations.

## 5. Conclusion

We proposed a novel, systematic method to model the temporal variation of a time series based on segmented linear regression. Its application to a real healthcare intervention demonstrated the method's ability to identify and describe trends over the study period, without bias to the intervention. We described a mathematically rigorous technique to determine whether trends are significant. The method offers a reliable tool in evaluating interventions and in detecting change, improving the information that can be drawn from retrospective studies.

## Figures and Tables

**Table 1:** A tabular illustration of the results of *F*-tests between models.

	Plateau	Line	Line⟷plateau
Line	*p*_line/plateau_		
Line⟷plateau	*p*_line-plateau/plateau_	*p*_line-plateau/line_	
Line-line	*p*_line-line/plateau_	*p*_line-line/line_	*p*_line-line/line-plateau_

The best model is the one that has a row of uninterrupted significant *p* values (*p* < 0.05) stretching furthest to the right. In the case of a tie, the lower-order model is preferred. If no significant *p* values feature in the table, the plateau is chosen as the best model.

**Table 2:** Patient demographics before and after implementation of the HFU.

Factor	Pre-HFU (*n*=2176)	Post-HFU (*n*=671)	*p* value
Age	84	85	0.0635
Gender	F:1575 M:601	F:477 M:194	0.5463
Pathological fracture	59	6	**0.0091**
ASA grade (1, 2, 3, 4, 5)	91, 574, 1178, 302, 3	12, 201, 378, 76, 1	**0.0094**
Reoperation	31	15	0.2035

Significant *p* values are in bold.

## Data Availability

The data used to support the findings of this study are available from the corresponding author upon request.

## Appendix

### A. Derivation of the Least Squares Parameters for the Line-Plateau and Plateau-Line Models

The line-plateau model is a straight line *y*_*i*_ *=* *m*_*k*_*t*_*i*_ *+* *c*_*k*_ for *i* *=* 1 to *k* adjoining to a plateau *y*_*j*_ *=* *Y*_k_ for *j* *=* *k* + 1 to *n*. *m*_*k*_ and *c*_*k*_ are the gradient and *y*-axis intercept of the straight line, respectively, and *Y*_*k*_ the value of the plateau. For each *k* from *k* = 1 to *n*, parameters *m*_*k*_ and *c*_*k*_ are found so as to minimise *R*, the sum of the squares of residuals:

(A.1) $$\begin{array}{c} R=\overset{n}{\underset{i=1}{\sum }}{\left(y_{i}-{\hat{y}}_{i}\right)}^{2}=\overset{k}{\underset{i=1}{\sum }}{\left(y_{i}-m\cdot t_{i}-c\right)}^{2}+\overset{n}{\underset{i=k+1}{\sum }}{\left(y_{i}-m\cdot t_{k}-c\right)}^{2}, \end{array}$$

where *y*_*i*_ are the ordinates of the points of the data series (*t*_*i*_, *y*_*i*_) and ${\hat{y}}_{i}$ are the predicted values of the model *y*_*i*_=*m* · *t*_*i*_+*c*, *i*=1  to  *k* and *y*_*i*_=*Y*, *i*=*k*+1  to  *n*.

Least squares fitting requires that ∂*R*/∂*m*=0 and ∂*R*/∂*c*=0 which together give the following matrix equation:

(A.2) $$\begin{array}{c} \left(\begin{array}{cc} A & B\\ B & n \end{array}\right)\left(\begin{array}{c} m\\ c \end{array}\right)=\left(\begin{array}{c} E\\ F \end{array}\right), \end{array}$$

where *A*=∑_*i*=1_^*k*^*t*_*i*_^2^+(*n* − *k*)*x*_*k*_^2^, *B*=∑_*i*=1_^*k*^*t*_*i*_+(*n* − *k*)*xt*_*k*_, *E*=∑_*i*=1_^*k*^*t*_*i*_*y*_*i*_+*xt*_*k*_ · ∑_*i*=*k*+1_^*n*^*y*_*i*_, and *F*=∑_*i*=1_^*n*^*y*_*i*_.

Equation (A.2) can be solved by linear algebra to yield values for *m* and *c* while *Y* (the plateau) is a constant value obtained from *Y* = *m · t*_*k*_ + *c*.

The best line-plateau model is that which yields a minimum value of *R* for different *k* (*k* = 1 to *n*):

$\left[m_{k},c_{k},k\right]=\underset{k}{\text{min}}\;\;\left[\sum_{i}{\left(y_{i}-{\hat{y}}_{i}\right)}^{2}\right]$; the derivation of the plateau-line model is very similar. Here a plateau *y*_*i*_=*c*, *i*=1  to  *k* adjoins a straight line. *y*_*i*_=*m* · (*t*_*i*_ − *t*_*k*_)+*c*, *i*=*k*+1  to  *n*.

Then, equation (A.2) becomes

(A.3) $$\begin{array}{c} R=\overset{k}{\underset{i=1}{\sum }}{\left(y_{i}-c\right)}^{2}+\overset{n}{\underset{i=k+1}{\sum }}{\left[y_{i}-m\cdot \left(t_{i}-t_{k}\right)-c\right]}^{2}. \end{array}$$

Least squares fitting requires that ∂*R*/∂*m*=0 and ∂*R*/∂*c*=0 which yields the following matrix equation:

(A.4) $$\begin{array}{c} \left(\begin{array}{cc} G & H\\ H & n \end{array}\right)\left(\begin{array}{c} m\\ c \end{array}\right)=\left(\begin{array}{c} L\\ F \end{array}\right), \end{array}$$

where *G*=∑_*i*=*k*+1_^*n*^*t*_*i*_^2^ − 2*t*_*k*_ · ∑_*i*=*k*+1_^*n*^*t*_*k*_+(*n* − *k*)*t*_*k*_^2^, *H*=∑_*i*=*k*+1_^*n*^*t*_*i*_ − (*n* − *k*)*t*_*k*_, *L*=∑_*i*=*k*+1_^*n*^*t*_*i*_*y*_*i*_ − *t*_*k*_ · ∑_*i*=*k*+1_^*n*^*y*_*i*_, and *F*=∑_*i*=1_^*n*^*y*_*i*_.

Equation (A.4) can be solved by linear algebra to yield values for the plateau *c* and the slope of the adjoining straight line *m* that joins the plateau at the *k*th data point. Once all values of *k* have been tried, the best plateau-line model is the one which yields a minimum value of *R* for different values of *k* (*k* = 1 to *n*):

(A.5) $$\begin{array}{c} \left[m_{k},c_{k},k\right]=\underset{k}{\text{min}}\;\;\left[\underset{i}{\sum }{\left(y_{i}-\;\;Y_{k}\right)}^{2}+\underset{j}{\sum }{\left(y_{j}-{\hat{y}}_{i}\right)}^{2}\right]. \end{array}$$

### B. Derivation of the Least Squares Parameters for the Line-Line Model

In the line-line model, a first straight line with equation *y*_*i*_ *=* *m*1_*k*_*t*_*i*_ *+* *c*_*k*_ (*i* *=* 1 to *k*) adjoins a second straight line with equation *y*_*j*_ *=* *m*2_*k*_ (*t*_*j* _– *t*_*k*_) *+* *y*_*k*_ (*j* *=* *k + *1 to *n*).

For each value of *k*, parameters *m*1, *c*, and *m*2 are found that minimise R:

(B.1) $$\begin{array}{c} R=\overset{k}{\underset{i=1}{\sum }}{\left(y_{i}-m1\cdot t_{i}-c\right)}^{2}+\overset{n}{\underset{i=k+1}{\sum }}{\left(y_{i}-m2\cdot \left(t_{i}-t_{k}\right)-m1\cdot t_{k}-c\right)}^{2}. \end{array}$$

Least squares fitting requires that ∂*R*/∂*m*  1=0, ∂*R*/∂*m*  2=0 and ∂*R*/∂*c*=0 which results in the following linear equation:

(B.2) $$\begin{array}{c} \left(\begin{array}{ccc} B & Q & n\\ S & T & Q\\ U & S & B \end{array}\right)\left(\begin{array}{c} m1\\ m2\\ c \end{array}\right)=\left(\begin{array}{c} F\\ Z\\ E \end{array}\right), \end{array}$$

where *B* = ∑_*i*=1_^*k*^*t*_*i*_+(*n* − *k*)*t*_*k*_, *Q*=∑_*i*=*k*+1_^*n*^*t*_*i*_ − (*n* − *k*)*t*_*k*_, *S*=*t*_*k*_∑_*i*=*k*+1_^*n*^*t*_*i*_(*n* − *k*)*t*_*k*_^2^, *T*=∑_*i*=*k*+1_^*n*^*t*_*i*_^2^+(*n* − *k*)*t*_*k*_^2^ − 2*t*_*k*_∑_*i*=*k*+1_^*n*^*xt*_*i*_, *U*=∑_*i*=*k*+1_^*n*^*t*_*i*_^2^+(*n* − *k*)*t*_*k*_^2^, *F*=∑_*i*=1_^*n*^*y*_*i*_, *Z*=∑_*i*=1_^*k*^*t*_*i*_*y*_*i*_ − *t*_*k*_ · ∑_*i*=*k*+1_^*n*^*y*_*i*_, and *E*=∑_*i*=1_^*k*^*t*_*i*_*y*_*i*_+*t*_*k*_ · ∑_*i*=*k*+1_^*n*^*y*_*i*_.

Equation (B.2) can be solved using linear algebra to yield values for *m*1, *m*2, and *c* and the process is repeated for all *k* to find the set of values [*m*1_*k*_, *c*_*k*_, *k*,  *m*2_*k*_] that yields the *s* minimum value of *R* for *k* = 1 to *n.*

(B.3) $$\begin{array}{c} \left[m1_{k},c_{k},k,m2_{k}\right]=\underset{k}{\text{min}}\left[\overset{k}{\underset{i=1}{\sum }}{\left(y_{i}-\;\;{\hat{y}}_{i}\right)}^{2}\right]. \end{array}$$
